# Overexpression of P-glycoprotein, MRP2, and CYP3A4 impairs intestinal absorption of octreotide in rats with portal hypertension

**DOI:** 10.1186/s12876-020-01532-4

**Published:** 2021-01-06

**Authors:** Xiaoyu Sun, Shunxiong Tang, Binbin Hou, Zhijun Duan, Zhen Liu, Yang Li, Shoucheng He, Qiuming Wang, Qingyong Chang

**Affiliations:** 1 Department of Gastroenterology, First Affiliated Hospital of Dalian Medical University, Dalian, 0086-116011 China; 2 Department of Invasive Technology, Affiliated Zhongshan Hospital of Dalian University, Dalian, China; 3 Department of Dermatology, Second Affiliated Hospital of Dalian Medical University, Dalian, China; 4 Department of Clinical Pharmacology, College of Pharmacy, Dalian Medical University, Dalian, China; 5 Department of Breast Surgery, Hospital of Chinese Medical University, Liaoning Provincial Cancer Institute and Hospital, Shenyang, China; 6 Department of Neurosurgery, Affiliated Zhongshan Hospital of Dalian University, Dalian, China

**Keywords:** P-glycoprotein, Multidrug resistance-associated protein 2, Cytochrome P450 3A4, Octreotide, Intestinal absorption, Portal hypertension

## Abstract

**Background:**

Portal hypertension (PH) is the main cause of complications and death in liver cirrhosis. The effect of oral administration of octreotide (OCT), a drug that reduces PH by the constriction of mesenteric arteries, is limited by a remarkable intestinal first-pass elimination.

**Methods:**

The bile duct ligation (BDL) was used in rats to induce liver cirrhosis with PH to examine the kinetics and molecular factors such as P-glycoprotein (P-gp), multidrug resistance-associated protein 2 (MRP2) and cytochrome P450 3A4 (CYP3A4) influencing the intestinal OCT absorption via in situ and in vitro experiments on jejunal segments, transportation experiments on Caco-2 cells and experiments using intestinal microsomes and recombinant human CYP3A4. Moreover, RT-PCR, western blot, and immunohistochemistry were performed.

**Results:**

Both in situ and in vitro experiments in jejunal segments showed that intestinal OCT absorption in both control and PH rats was largely controlled by P-gp and, to a lesser extent, by MRP2. OCT transport mediated by P-gp and MRP2 was demonstrated on Caco-2 cells. The results of RT-PCR, western blot, and immunohistochemistry suggested that impaired OCT absorption in PH was in part due to the jejunal upregulation of these two transporters. The use of intestinal microsomes and recombinant human CYP3A4 revealed that CYP3A4 metabolized OCT, and its upregulation in PH likely contributed to impaired drug absorption.

**Conclusions:**

Inhibition of P-gp, MRP2, and CYP3A4 might represent a valid option for decreasing intestinal first-pass effects on orally administered OCT, thereby increasing its bioavailability to alleviate PH in patients with cirrhosis.

## Background

Portal hypertension (PH) is defined by a portal vein pressure greater than 10 mmHg and is the main cause of complications and death in patients with liver cirrhosis. So far, standard treatments against PH are mainly aimed at treating varicosis and variceal bleeding. The treatment stratification is based on the action on different clinical PH stages. The purpose of this treatment in PH patients with cirrhosis without varicose veins is to prevent both varicosis (pre-primary prophylaxis), and variceal bleeding in patients with gastroesophageal varices (primary prophylaxis). Under the condition of acute variceal bleeding (AVB), the treatment consists of hemostasis and prevention of early re-bleeding, and it should prevent delayed re-bleeding in AVB survivors (secondary prophylaxis) [1]. Early control of PH is recommended in clinical practice; however, side effects and poor efficacy of current oral medicines limit the success of PH therapy. Non-selective beta-blockers (NBSS) are recommended as a standard therapy in the primary and secondary prophylaxis against variceal bleeding in patients with cirrhosis and high risk esophageal varices (EVs) [2]. Although several studies evaluated the efficacy of NBSS in the inhibition of the development or aggravation of varices, results are controversial; no recommendation is available for patients with cirrhosis without varices or with low risk of small varices [1]. NBSS reduce the probability of bleeding from 40%-50% (without any therapy) to 25% over a 2-year period, but the efficiency is decreasing with time. Moreover, not all patients exhibit a hemodynamic response, and these non-responsive patients have a risk of bleeding similar to the one of non-treated patients. If the hepatic vein pressure gradient (HVPG) is not measured, clinical protocols should be used for titrating NBSS (reduction of resting heart rate to 55 beats/min or 25% of the baseline) [3]. Moreover, at least one third of the patients are unresponsive, have contraindications or cannot tolerate NBSS therapy [4, 5]. Thus, NBSS effect can be limited by its side effects.

Hepatic and intestinal first-pass effect affects the oral absorption and bioavailability of medicines. Little is known about how PH and the vascular changes often associated with this condition affect drug absorption. Previous studies showed that mal-absorption or enhanced absorption of drugs can occur depending on the extent of different lesions in liver cirrhosis because of the changes in the expression or activity of drug transporters or metabolic enzymes in liver or intestine [6–16], while venous shunt formation in PH may effectively increase the absorption of various drugs [17]. Hepatic first-pass effects are reduced during PH due to the increased collateral circulation.

The ATP-driven drug efflux pump P-glycoprotein (P-gp, MDR1-gene product, mdr1a and mdr1b subtypes) [18, 19] and the multidrug resistance-associated protein 2 (MRP2, mrp2-gene product) [20, 21] largely influence drug uptake, distribution, and excretion in the intestine, liver and kidney. Octreotide (OCT), an octapeptide that pharmacologically mimics natural somatostatin, can reduce portal pressure and portal blood flow by the induction of selective splanchnic vasoconstriction [22]. These effects are partly mediated by the inhibition of the release of vasoactive substances including glucagon, which indirectly causes dilatation of the splanchnic vessels [23, 24]. OCT is widely used in the treatment of PH hemorrhage and related complications [24, 25]. Practice guidelines recommend OCT as an adjuvant therapy in the control of variceal hemorrhage, along with timely therapeutic endoscopy [26–28]. It is currently recommended that intravenous (i.v.) OCT should be started as soon as possible before the endoscopic therapy in suspected variceal hemorrhage [29]. OCT is as effective as terlipressin in patients with AVB [30]. In addition, OCT has few side effects, since only mild hyperglycemia and abdominal cramps have been reported. It is routinely used worldwide largely because of its excellent safety profile and ease of administration [3, 31], and it is a universally recognized drug that can reduce PH and control variceal bleeding, being the only available vasoactive drug in the United States [31]. Therefore, OCT may be considered as an ideal oral drug to decrease PH. According to the current PH treatment guidelines, it is expected that in the future, oral OCT, which is the topic this work focused on, will be used in pre-primary, primary, and secondary prophylaxis of variceal bleeding, except on AVB patients. Thus, our hope was to find an effective way to prevent PH development with an early, continuous, and lifelong strategy such as the treatment against hypertension. However, OCT exhibits low systemic bioavailability upon ingestion due to its polypeptide structure and its use is limited to parenteral administration. After subcutaneous or i.v. injection, OCT is rapidly and completely absorbed with few side effects [32]. Additionally, patients with liver cirrhosis have prolonged elimination of OCT with a greatly increased t_1/2_ and decreased total body clearance [33]. OCT is stable against enzymatic degradation in the stomach, meaning that it can overcome problems, to a certain extent, associated with the short biological half-life of therapeutically active peptides [34]. Thus, this drug may represent a valuable oral medicine for long-term use that can persistently decrease PH. Previous studies recommended the oral administration of a novel OCT formulation as an alternative to parenteral OCT treatment in patients with acromegaly [35, 36]. As regard its chronic use, OCT long-acting release (LAR) can be administered once monthly by intramuscular injection [37]. Since OCT-LAR, such as lanreotide, is similar to OCT regarding pharmacology, clinical actions, and most adverse effects [38], it remains unclear whether OCT-LAR can be safely used for PH in patients with compensated cirrhosis [39]. In addition, the appropriate dose and schedule required for long-term therapy of OCT-LAR are not clear [40]. The safety profile, pharmacokinetics, and pharmacodynamics of OCT-LAR have not been carefully studied in patients with cirrhosis [39, 41]. In addition, OCT-LAR costs much more than conventional OCT, thus resulting in a huge economic burden for patients [40]. Moreover, OCT-LAR injection should be performed at the hospital, while the hope of this work is to realize a therapy involving the oral administration of OCT performed at home, which is more convenient for patients.

OCT absorption across the blood–brain barrier [42] and into renal proximal tubules [21] is prevented by P-gp and MRP2. In addition, cytochrome P450 3A4 (CYP3A4, cyp3A1-gene product in rats), one of the most important CYP enzymes in the small intestine, also functions as a barrier against drugs, including polypeptides [43]. The substrates of P-gp, MRP2, and CYP3A4 often overlap, leading to synergistic effects on the same substrate. Thus, our hypothesis is that P-gp, MRP2, and CYP3A4 might contribute to the intestinal first-pass effect on OCT, thus limiting its oral absorption. Therefore, the aim of the current study was to determine whether P-gp and MRP2 affect OCT transport, and whether CYP3A4 affects OCT metabolism in the intestine of rats with cirrhosis and PH, with the intention of improving the intestinal absorption of OCT and optimizing oral therapeutic strategies for decreasing PH.

## Methods

### Animal care

Male Sprague Dawley rats (7 weeks ± 2 days, weighing 200–220 g) were purchased from the Experimental Animal Center at Dalian Medical University (Liaoning, China). This study was carried out in strict accordance with the International Council for Laboratory Animal Science. All animal experiments were conducted in accordance with the protocols approved by the Experimental Animal Ethical Committee of Dalian Medical University (No. L20180301). All surgeries were performed under fully anesthesia with ether (Laboratory regent, 5 mL inhalation for 30–45 s) to obtain well anesthetized rats to allow experiments as previously reported [44, 45]. The term “well anesthetized” is used when the corneal reflex and pain reflex in rats are disappeared. The effect of anesthesia began to fade after 20 min, thus, the rats were anesthetized again with an additional dose of anesthetic. Animals remained anesthetized during the whole experiment. After the establishment of PH rat models, the rats were anesthetized every other hour for 24 h to reduce the postoperative pain and were kept under observation for potential abnormalities. All the necessary efforts were made to reduce the number of dropouts. All animal experiments were carried out after PH rats were successfully established, and no dropout was observed during anesthesia or each experiment. Each experiment was successfully finished. The successful rate of the model in our research was approximately 70%. Animals were euthanized through cervical dislocation under anesthesia.

### Animal model

Rats were housed at room temperature (24–26 °C) and relative humidity of 60%-65% in a specific pathogen-free environment. They had free access to water and were fed with a chow diet for 3 days prior to any experiment. The animals were fasted for 12 h with water available ad libitum prior to the pharmacokinetic experiments. Biliary cirrhosis with PH was induced via bile duct ligation (BDL) as previously described [46]. The bile duct was isolated and double-ligated using 3–0 silk sutures. The abdominal wall and skin were closed using 4–0 silk sutures, and the antibiotic gentamicin (30 mg/kg, 0.3 mL) was administered through an intramuscular injection. Rats were allowed to recover with unrestricted access to food and water for a 4-week period after surgery. In this study, 80 rats were randomly selected from a total of 100 to build the PH rat model, and 57 were successfully established. Then, 48 of them were randomly selected and divided into the respective PH experimental groups (n = 4 or 6). The remaining PH rats were kept for a further potential use. Portal vein pressure was measured in PH rats before each experiment, resulting in an average value of 15.56 ± 2.36 mmHg. Administration methods and measurements in each group are further specified in the appropriate sections of the text. The jejunum was the intestinal tract investigated in the present study, since it is the main site of OCT absorption [47]. Sample size in each group was determined according to research articles relevant to this study.

### Pharmacokinetic interaction study

#### In situ jejunal perfusion technique

The experimental procedure was performed as previously reported. Briefly, the mean length of the proximal jejunum (approximately 2 cm distal to the ligament of Treitz) was 10 ± 1 cm [48]. An oxygenated Krebs–Ringer bicarbonate buffer (KRB) solution (pH 7.4) containing 0.5 mM MgCl_2_, 4.5 mM KCl, 120 mM NaCl, 0.7 mM Na_2_HPO_4_, 1.5 mM NaH_2_PO_4_, 1.2 mM CaCl_2_, 15 mM NaHCO_3_, and 10 mM glucose was perfused at a flow rate of 0.4 mL/min using a peristaltic pump through an inlet tube kept at 37 °C. After a 30-min equilibration period, the following drugs (dissolved in KRB) were administered: (1) 10 µM OCT (99% purity, Chengdu Xinlinbang Bio-pharmaceutical Co.) (Group A, control group, n = 4); (2) 10 µM OCT plus 1 mM verapamil hydrochloride (Sigma-Aldrich, St. Louis, MO) (Group B, n = 4); (3) 10 µM OCT plus 1 mM probenecid (Sigma-Aldrich, St. Louis, MO) (Group C, n = 4); and (4) 10 µM OCT plus 1 mM verapamil hydrochloride plus 1 mM probenecid (Group D, n = 4). Portal vein blood (200–300 µl) was collected at 5, 10, 15, 30, 45, and 60 min for OCT determination via LC–MS/MS. The AUC was calculated using the integral method. These experiments were performed both in healthy and PH rats; the latter was called Group A1, B1, C1, and D1 (*n* = 4 per group).

#### In vitro everted intestinal sac preparation

Rat jejunum (10 ± 1 cm) was transected approximately 2 cm distal to the ligament of Treitz [48]. The empty sac was filled with 1 mL KRB (pH 7.4) and placed in incubation medium (mucosal solution) containing (1) 10 µM OCT (Group A, control group); (2) 10 µM OCT plus 1 mM verapamil hydrochloride (Group B); (3) 10 µM OCT plus 1 mM probenecid (Group C); or (4) 10 µM OCT plus 1 mM verapamil hydrochloride plus 1 mM probenecid (Group D) (n = 4 for each group). Samples of the lumen of the sac (50 µl) were collected at 10, 15, 30, 45, 60, 90, and 120 min for OCT determination via LC–MS/MS. The AUC was calculated using the trapezoid method. These experiments were performed in both control and PH rats; the latter was called Group A1, B1, C1, and D1 (n = 4 per group).

### Caco-2 permeability assay

#### Cell culture

Caco-2 cells (purchased from the Type Culture Collection of the Chinese Academy of Sciences, Shanghai, China) at passage 30 to 33 were used in this study. Cells were routinely cultured as previously described with minor modifications [49]. Cells were seeded onto Transwell inserts in 12-well dishes at a density of 5 × 10^5^ cells/well (#3401; 0.4 µm pore size, growth area of 1.13 cm^2^, Corning, NY, USA), and the medium was replaced with D-Hanks solution every other day except for the treatment days [50]. No sign of Caco-2 cell death was observed at OCT concentrations of 1, 10, and 50 µM as determined by the LDH-cytotoxicity assay (data not shown).

Cell monolayer integrity was evaluated through the transepithelial electrical resistance (TEER) using a Millicell ERS resistance system (Millipore, Bedford, MA, USA) at 37 °C. Each TEER value was corrected for background resistance determined on an extracellular matrix-coated cell culture insert without cells. Monolayers that exhibited a TEER value > 350 Ω·cm^2^ on the 21st day of cell culture were used for the transport study.

#### Transport study

Caco-2 transport study was performed as previously described [50]. Briefly, apical to basolateral (A to B) or basolateral to apical (B to A) transport assay started after gentle removal of the HBSS buffer in the donor compartment and addition of 1.4 mL (1) OCT (10 µM); (2) OCT (10 µM) plus the specific P-gp inhibitor rhodamine 123 (1 mM; Sigma-Aldrich, St. Louis, MO); or (3) OCT (10 µM) plus probenecid (1 mM). A to B assay was performed to determine the extent of OCT absorption, while B to A assay was performed to measure OCT secretion. As regard A to B assay, the apical (upper) chamber was filled with 1.6 mL test drug solution in HBSS, while the receiver basolateral (lower) compartment was filled with 0.6 mL HBSS alone. Conversely, in B to A assay the upper and lower chambers were filled with 0.6 mL HBSS and 1.6 mL test drug, respectively. A small aliquot (0.05 mL) of HBSS was collected to determine the OCT loading concentration C_0_. The TEER value was determined at different incubation times to verify the integrity of the monolayer after buffer change. A total amount of 100 µL was collected from the receiver compartment at 5, 15, 30, 60, and 90 min to determine the OCT concentration by LC–MS/MS. The concentration kinetic of OCT uptake was measured at pH 6.0 over a concentration range of 1 to 50 µM at 37 °C, as described above. The apparent permeability coefficient (P_app_, cm/s) of OCT across the Caco-2 monolayer was calculated using the following equation:

$${\text{P}}_{{{\text{app}}}} =\Delta {\text{A}}_{{\text{R}}} /\Delta t/{\text{Area}} \times 60 \times {\text{C}}_{0}$$

where ΔA_R_ is the amount of drug accumulated in the receiver compartment during the interval time (Δt) and Area is the surface area of the filter/membrane of the Transwell (cm^2^). The permeability direction rate (PDR) was calculated by the ratio of P_app_ in the B to A direction versus that in the A to B direction, as follows:

$${\text{PDR}} = {\text{P}}_{{{\text{app}}\,({\text{B}}\,{\text{to}}\,{\text{A}})}} /{\text{P}}_{{{\text{app}}\,({\text{A}}\,{\text{to}}\,{\text{B}})}}$$

#### OCT metabolism by CYP3A in rat microsomal preparation

Microsomes from the small intestine of healthy rats and PH rats were obtained as previously described [51], and their protein concentration (0.53 ± 0.05 mg/mL and 0.52 ± 0.06 mg/mL total protein, respectively) was measured using the BCA method (Beijing Solarbio Science & Technology Co., Ltd). The reaction mixture (500 µL) containing 0.4 mg intestinal microsome protein, 100 mM potassium phosphate buffer (PPB; pH 7.4), a NADPH-generating system (3.3 mM MgCl_2_, 1.3 mM β-NADP^+^, 3.3 mM glucose 6-phosphate, 0.4 U/mL glucose-6-phosphate dehydrogenase), and OCT (1000 ng/mL), was incubated at 37 °C for 30 min with or without ketoconazole (10 µM, Sigma-Aldrich, St. Louis, MO). CYP3A activity was measured using a fluorescent quantitative detection kit (Genmed Scientifics Inc., U.S.A.) with the NADPH regenerating system after pre-incubation at 37 °C for 0, 2, 10, 20 or 30 min. In addition, the effect of OCT pre-incubation on CYP3A inactivation was determined by pre-incubating OCT (1000 ng/mL) with microsomes, prepared in PPB, for 0, 2, 10, 20 or 30 min at 37 °C in the presence of a NADPH regenerating system. The organic phase was evaporated to dryness and the OCT content in the residue was measured by LC–MS/MS.

Microsomes prepared from healthy rats were equally divided into 2 groups: (1) OCT (Group N + OCT) and (2) OCT + ketoconazole (Group N + OCT + K). Similarly, microsomes prepared from PH rats were divided into two groups: (1) OCT (Group PH + OCT) and (2) OCT + ketoconazole (Group PH + OCT + K) (n = 6 in each group). The control group (Group OCT; n = 6) contained OCT without microsomes. Each sample was tested 3 times.

#### OCT metabolism by recombinant human cytochrome P450 3A4

All incubations were performed at 37 °C in a water bath. Recombinant human cDNA-expressing P450 isozyme 3A4 (BD Gentest Supersomes) was carefully thawed on ice prior to each experiment. The reaction mixture (200 µL) contained 10 µL human recombinant CYP3A4, 2 µL OCT (at different concentrations; see below), 168 µl PPB (100 mM), and 20 µL NADPH (1 mM). After a 5-min pre-incubation at 37 °C, the reaction was started by the addition of NADPH and ended 30 min later by the addition of 500 µL methanol. Control samples without NADPH and samples without OCT were also included. At the end of each reaction, samples were centrifuged at 12,000*g* for 5 min at 4 °C to precipitate the proteins, and the supernatant was subjected to LC–MS/MS analysis to determine the OCT content. Each sample was tested 4 times. CYP3A4 activity with and without OCT incubation was assessed using a fluorescent quantitative detection kit.

To determine the optimal experimental conditions, different incubation times such as 5, 10, 15, 20, and 30 min were used together with a CYP3A4 concentration of 2.5 mg/mL with an OCT concentration of 100 mM to determine the most effective incubation time within a linear range. To determine the optimal protein concentration of recombinant CYP3A4 within a linear range, different recombinant CYP3A4 concentrations were used, such as 1.25, 2.5, 5, 10, and 20 mg/mL using an incubation time of 10 min and an OCT concentration of 100 mM. Based on the optimized CYP3A4 protein concentration at different reaction times, different OCT concentrations (20, 50, 100, 200, and 400 mM) were added to the reaction mixture and the experiment was then performed as described above.

### P-gp, MRP2, and CYP3A4 expression in the rat intestinal mucosa

Upper jejunum samples from normal (Group N, n = 3) and PH (Group PH, n = 3) rats were used to assess the expression of P-gp, MRP2, and CYP3A4 by reverse transcription-polymerase chain reaction (RT-PCR), western blotting, and immunohistochemistry.

#### RT-PCR

RT-PCR was performed as previously described [48]. Jejunum tissue samples were stored in RNA stabilizer (DaLian Pauley Shield Bio-Engineering Co., Ltd) followed by rapid freezing to prevent RNA degradation. Total RNA was extracted from each perfused sample using the TRIzol reagent (Invitrogen, Shanghai, China) according to the manufacturer’s protocol and was analyzed by ultraviolet spectrophotometry. A 2-step RT-PCR was performed using the Taq DNA polymerase kit (Takara, Dalian, China) according to the provided protocol, and cDNA was subsequently amplified using a GeneAmp PCR system (Techne TC512, UK). RNA samples (500 ng) were reverse transcribed and immediately amplified by PCR. Reverse transcription was performed for 10 min at 30 °C, followed by 30 min at 42 °C, and the samples were subsequently heated for 5 min at 95 °C to terminate the reaction. β-actin (5′-GGGACCTGACAGACTACCTC-3′ forward and 5′- GACAGCACTGTGTTGGCATAG-3′ reverse) was used as the internal control. The primers (Takara, Dalian, China) used where the following: mdr1a-F, 5′-GATGGAATTGATAATGTGGACA-3′; mdr1a-R 5′-AAGGATCAGGAACAATAAA-3′; mdr1b-F, 5′-GAAATAATGCTTATGAATCCCAAAG-3′; mdr1b-R, 5′-GGTTTCATGGTCGTCGTCTCTTGA-3′; mrp2-F, 5′-ACCTTCCACGTAGTGATCCT -3′; mrp2-R, 5′-ACCTGCTAAGATGGACGGTC-3′; cyp3A1-F, 5′-GAGGAGTAATTTGCTGACAGAACCTGC-3′; cyp3A1-R, 5′-CCAGGAATCCCCTGTTTCTTGAA-3′(Additional file 1: Table S1). Quantity One (version 4.40) software (Bio-Rad, CA, USA) was used to analyze the band density on each gel. β-actin mRNA expression was used to normalize the expression of the target genes.

#### Western blot

Upper jejunum samples were frozen and stored in radio-immunoprecipitation (RIPA) buffer. Western blotting was performed as previously described [32]. Mouse P-gp monoclonal antibody C219 (1:75) (Thermo Scientific, Pierce Biotechnology, USA), rabbit-anti-mouse MRP2 monoclonal antibody (1:100), rabbit-anti-mouse CYP3A4 polyclonal antibody (1:1000) (Abcam, Cambridge, MA, USA), and a β-actin monoclonal antibody (1:1000) (Beijing Zhong Shan Golden Bridge Biological Technology CO., LTD) were used. Anti-rabbit (1:800) or anti-mouse (1:1000) horseradish peroxidase-conjugated antibody (Beyotime Institute of Biotechnology, China) was used as the secondary antibody for the final protein detection. Protein bands were visualized and photographed under transmitted ultraviolet light for semiquantitative measurements based on band densitometry. β-actin was used as the loading control.

#### Immunohistochemistry

**Fig. 1:**
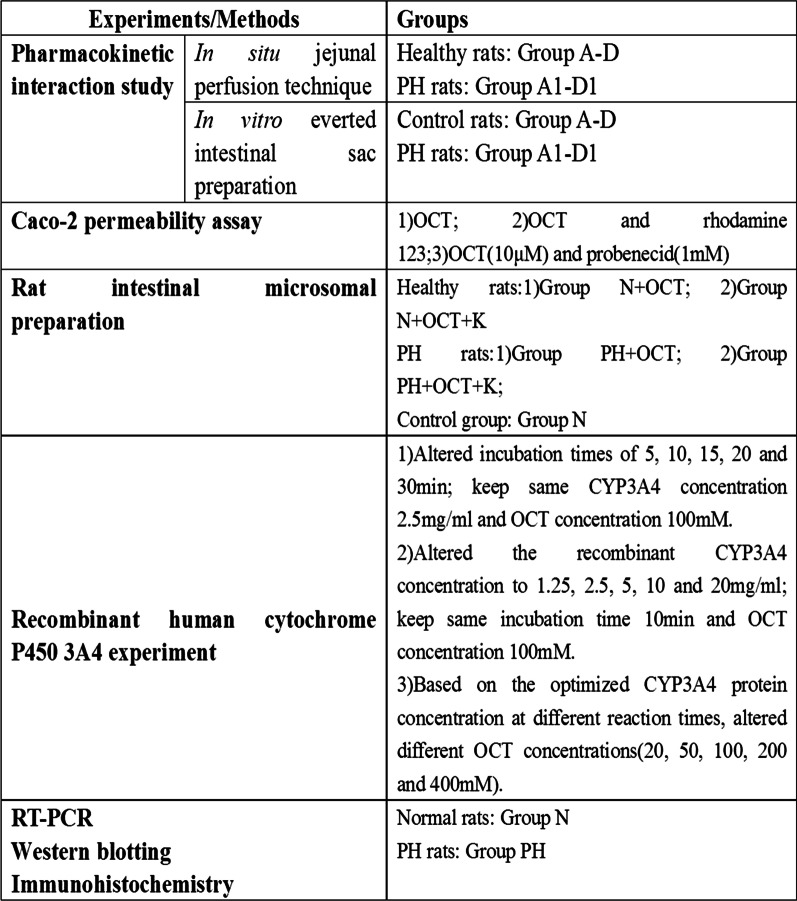
Graphical explanation of the performed experiments/methods together with the respective groups

Upper jejunum samples were prepared for the immunohistochemical staining as previously described [46]. Primary P-gp, MRP2, and CYP3A4 antibodies were the same as those used to perform western blotting, and used at the dilution of 1:58, 1:100 and 1:70, respectively. Phosphate-buffered saline replaced the primary antibodies in the negative controls. Yellow staining of membranes and cytoplasm indicated P-gp and MRP2 positivity, while the yellow staining only in the membrane indicated CYP3A4 positivity A total of 5 high-power microscopic fields per slide were randomly selected for signal quantification. Cell staining was scored between 1 and 4, depending on the staining intensity. The final score was defined as the staining intensity × percentage of positive cells [52]. The mean score of 5 fields was used to compare the 5 groups. Figure 1 shows a graphical explanation of the performed experiments/methods together with the respective groups.

### Sample preparation for LC − MS/MS analysis

Frozen samples were thawed at room temperature prior to their preparation. A 200-µL aliquot of cell lysate containing 1 mL Tris, 99 mL D-Hank’s, and 200 µL methyl cyanide was mixed with the sample obtained from Caco-2 experiments. The mixture was then vortexed for 1 min and centrifuged at 12,000×*g* for 10 min. The supernatant was transferred to a clean glass tube and evaporated to dryness at 40 °C under nitrogen. A 10-μL aliquot was injected for LC − MS/MS analysis. A total of 200 μL methanol was added to a 50-μL sample of plasma, and the mixture was vortexed and centrifuged as described above to remove precipitated proteins. A total of 200 μL supernatant was transferred and evaporated as described for the Caco-2 samples. The residue was then diluted with 100 μL mobile phase and an aliquot of 10 μL was used for LC − MS/MS analysis.

### OCT content by LC–MS/MS

An Agilent LC system (Agilent HP1200, Agilent Technology Inc., Palo Alto, CA, USA) was used to measure the OCT content. Isocratic chromatographic separation was performed using a Hypersil BDS-C18 column (150 mm × 4.6 mm i.d., 5 µm; Dalian Elite Analytical Instruments Co. Ltd., China) maintained at room temperature. A mixture of 40% methanol-60% water with 0.1% formic acid (60:40, v/v) was used as the mobile phase at a flow rate of 0.5 mL/min. An API 3200 triple-quadrupole mass spectrometer (Applied Biosystems, Concord, Ontario, Canada) was operated with a Turbo Ionspray interface in positive ion mode. The ion spray voltage was set to + 4 psi, heater gas temperature to 500 °C, nebulizer gas (Gas 1) at 40 psi, heater gas (Gas 2) at 40 psi, curtain gas at 10 psi, and collision gas at 16 psi.

The declustering potential was set to 30 V for the analyses and IS. Multiple reaction monitoring (MRM) was employed for data acquisition. The optimized MRM fragmentation transition was m/z 510.2 → m/z 120.1, with collision energy of 50 eV for OCT. The dwell time for each transition was 200 ms. Data processing was performed using the Analyst 1.4.1 software package (Applied Biosystems).

### Statistical analysis

SPSS 11.5 software was used for statistical analysis. All measurements were expressed as mean ± SD. One-way analysis of variance (ANOVA) was performed to evaluate significant differences among multiple treatments for a specific parameter. A value of *p* < 0.05 was considered statistically significant.

## Results

### P-gp and MRP2 inhibition increases jejunal OCT absorption in situ

**Fig. 2:**
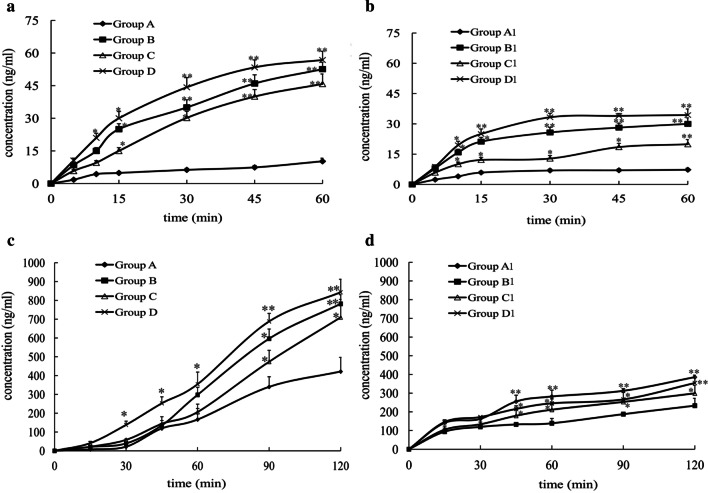
Effects of P-gp and MRP2 inhibition on the time profile of OCT transport in the intestine. **a** OCT absorption kinetics in in situ jejunal perfusion experiments in healthy rats; **b** OCT absorption kinetics in in situ jejunal perfusion experiments in PH rats; **c** OCT absorption kinetics in the everted intestinal sac preparation in healthy rats; **d** OCT absorption kinetics in the everted intestinal sac preparation in PH rats; Group A, control group; Group B, OCT plus verapamil hydrochloride; Group C, OCT plus probenecid; Group D, OCT plus verapamil hydrochloride plus probenecid. Results are expressed as mean ± S.D. (*n* = 4). *p < 0.05 versus control (Group A, Group A1), **p < 0.01 versus control (Group A, Group A1)

In situ jejunal perfusion was used to determine the effect of P-gp and MRP2 inhibitors on the absorption of OCT in healthy (Groups A-D) and PH (Groups A1-D1) rats. The OCT level in Group D (receiving OCT plus both verapamil hydrochloride, a P-gp inhibitor, and probenecid, an MRP2 inhibitor) was significantly increased compared with its level in the Group A, B, and C (*p* < 0.05, Fig. 2a). The OCT content in Group B (OCT plus verapamil hydrochloride) was also significantly increased compared with its content in the Group C (OCT plus probenecid) (*p* < 0.05). Similar results were obtained in PH rats (Fig. 2b). The OCT absorption was lower under each condition used in PH rats compared to its absorption in normal rats (*p* < 0.05). The AUC in Group B, C, and D was 521.08%, 418.95%, and 618.40% respectively, of the correspondent control (Group A; OCT only), while the AUC of Group B1, C1, and D1 was 384.61%, 236.28%, and 461.78% respectively, of the correspondent control group (Group A1). In turn, the AUC of group A1 was 96% of group A. These results indicated that P-gp and, to a lesser extent, MRP2 activity inhibited OCT absorption in situ. Moreover, these results indicated that intestinal OCT absorption was impaired in PH rats, suggesting that other mechanisms besides P-gp and MRP2 were potentially involved in this process.

### P-gp and MRP2 inhibition increases OCT uptake in the everted intestinal sac model

To confirm the involvement of P-gp and MRP2 in the intestinal OCT absorption and assess the difference between control and PH rats, the rat everted intestinal sac in vitro model was used. The OCT concentration in Group D was significantly increased compared with its concentration in Group A, B, and C (*p* < 0.05, Fig. 2c). Moreover, the OCT content in Group B was significantly increased compared with its content in Group C (*p* < 0.05). Similar results were observed in PH rats (Fig. 2d). Moreover, the OCT level in PH rats was significantly decreased compared with that in the corresponding control groups (*p* < 0.05). The AUC value in Group B, C, and D was 176.22%, 151.39%, and 220.63% respectively, of the correspondent control group (Group A). The AUC value in Group B1, C1, and D1 was 151.82%, 128.23%, and 165.33% respectively, of the correspondent control group (Group A1). The AUC of Group A1 was 21.54% of the one in Group A. Although clear differences in the degree of OCT transport were observed between these experiments and in situ jejunal perfusion experiments (probably due to the lack of physiological environment in the everted sac preparation), the above results confirmed that rats with PH had a reduced intestinal OCT absorption.

### Cell-based OCT permeability assay

A bi-directional Caco-2 cell permeability assay was further performed to examine the effect of P-gp and MRP2 inhibition on OCT absorption (i.e. apical to basolateral -A/B- transport and secretion -B/A- transport). The assay was performed using 1, 10, and 50 µM OCT in the presence or absence of rhodamine 123 (1 mM) and probenecid (1 mM), to inhibit P-gp and MRP2, respectively. Table 1 shows the measured permeability coefficient (P_app_) and flux ratio (permeability direction rate, PDR; B to A/A to B). Overall, the results indicated that P-gp and MRP2 effectively mediated OCT transport in Caco-2 cells, as demonstrated by the PDR > 1.5 indicating active drug transport. The determination of OCT at a dose of 1 μM was unreliable, as some sample points approached the quantitative limit of the triple-quadrupole mass spectrometer assay. In turn, 50 μM OCT was close to the saturation concentration, and it did not yield an accurate flux measurement. Reliable data were instead recorded with 10 μM OCT, roughly equivalent to a clinical dose of 80 mg [36]. The secretion (B to A flux) of OCT by Caco-2 monolayers was 4.4 times greater than its absorption (A to B flux) (Table 1). P_app (A-B)_ significantly increased when P-gp or MRP2 was inhibited, particularly when P-gp and MRP2 were simultaneously inhibited. In contrast, P_app (B-A)_ was significantly decreased by each individual inhibitor, and the effect was even stronger upon co-inhibition. These results were consistent with P-gp and MRP2 being predominantly located at the apical cell membrane [53], therefore acting to limit drug absorption.

**Table 1:** Effect of P-gp and MRP2 inhibition on OCT transport by Caco-2 cells

Inhibition		OCT 1 μM		OCT 10 μM		OCT 50 μM	
		A-B	B-A	A-B	B-A	A-B	B-A
MRP2	P_app_(× 10^−6^ cm/s)	0.007 ± 0.001	0.006 ± 0.001	0.149 ± 0.016^a^	0.31 ± 0.025^b^	0.353 ± 0.032	1.13 ± 0.097
	PDR(B-A/A-B)	0.9		2.1		3.2	
P-gp	P_app_(× 10^−6^ cm/s)	0.010 ± 0.001	0.012 ± 0.002	0.168 ± 0.018^a^	0.297 ± 0.024^b^	1.192 ± 0.072	1.040 ± 0.084
	PDR(B-A/A-B)	1.2		1.8		0.9	
MRP2 and P-gp	P_app_(× 10^−6^ cm/s)	0.022 ± 0.002	0.006 ± 0.001	0.368 ± 0.055^a,c^	0.193 ± 0.021^b,c^	1.725 ± 0.138	0.981 ± 0.082
	PDR(B-A/A-B)	0.3		0.5		0.6	
Control	P_app_(× 10^−6^ cm/s)	0.005 ± 0.001	0.021 ± 0.003	0.127 ± 0.015	0.565 ± 0.073^c^	0.23 ± 0.021	1.334 ± 0.160
	PDR(B-A/A-B)	4.1		4.4		5.8	

Results are expressed as mean ± SD; n = 4 per treatment

A-B, Apical-to-Basolateral; B-A, Basolateral-to-Apical

P_app_, permeability coefficient; PDR, permeability direction rate

^a^Indicates *p* < 0.05 for: P_app_ (A to B) of MRP2 and P-gp inhibition versus P_app_ (A to B) of MRP2 or P-gp inhibition; P_app_ (A to B) of P-gp inhibition versus control; and P_app_ (A to B) of MRP2 inhibition versus Control

^b^Indicates *p* < 0.05 for P_app_ (B to A) versus P_app_ (A to B) upon inhibition of MRP2 or P-gp and combined inhibition of MRP2 and P-gp

^c^Indicates *p* < 0.01 for: P_app_ (A to B) of MRP2 and P-gp inhibition versus Control; P_app_ (B to A) of MRP2 and P-gp inhibition versus control; and P_app_ (B to A) control versus P_app_ (A to B) control (control = 10 μM OCT)

### CYP3A-mediated OCT metabolism is increased in rat intestinal microsomes

**Fig. 3:**
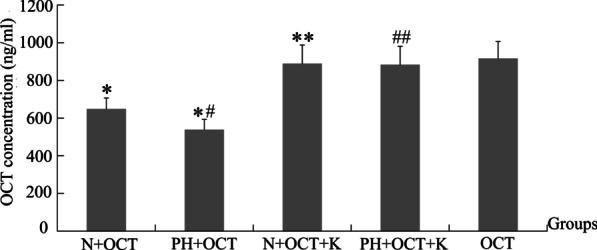
OCT metabolism by CYP3A in rat intestinal microsomes. CYP3A activity was measured using a fluorescent detection kit, and OCT content was determined at different times by LC–MS/MS. Results are expressed as mean ± S.D. (*n* = 6). *p < 0.01, Group N + OCT versus Group OCT, Group PH + OCT versus Group OCT; **p < 0.01, Group N + OCT + K versus Group N + OCT; #*p* < 0.05, Group PH + OCT versus Group N + OCT; ^##^*p* < 0.01, Group PH + OCT + K versus Group PH + OCT. Group OCT, OCT alone without microsomes; N, Normal microsomes; PH, PH microsomes; K, ketoconazole

OCT metabolism by CYP3A was assessed in intestinal microsomes isolated from healthy (N) and PH rats. CYP3A activity was approximately 280 pmol/(mg*min) in microsomes from normal rats, while its activity was approximately 350 pmol/(mg*min) in microsomes from PH rats. The OCT level was significantly lower in the PH + OCT group than in the N + OCT group (*p* < 0.05), and significantly and equally increased in both groups in the presence of the CYP3A inhibitor ketoconazole (*p* < 0.01). These results indicated that OCT was metabolized by CYP3A, and the expression or activity of this enzyme was increased in the intestinal tissue of rats with PH (Fig. 3).

### OCT is metabolized by recombinant human CYP3A4

**Fig. 4:**
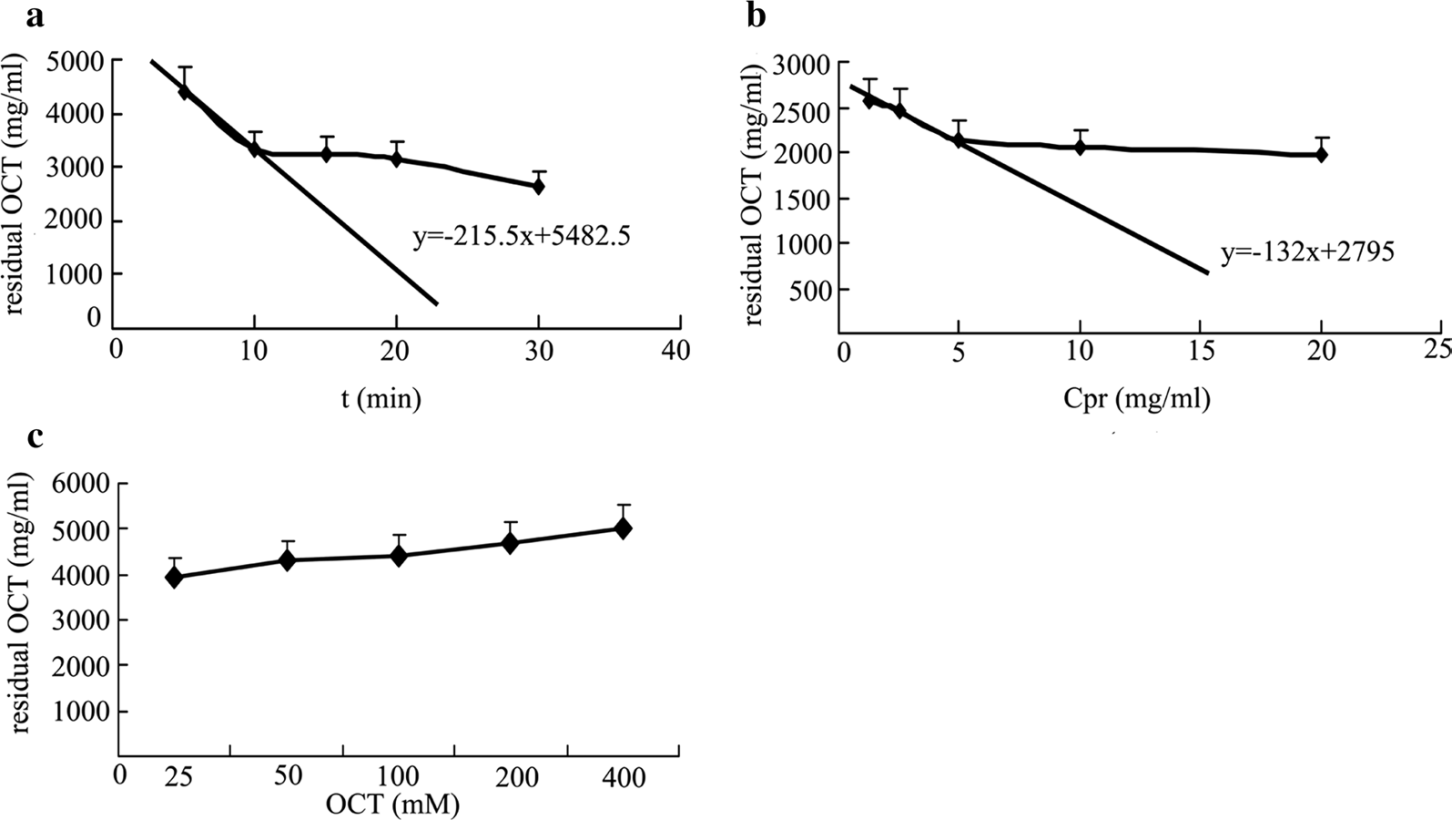
OCT metabolism by recombinant human CYP3A4. **a** Correlation between incubation time and residual OCT concentration; the most effective incubation time was 10 min. **b** Correlation between recombinant CYP3A4 and residual OCT concentration; the most effective CYP3A4 concentration was 2.5 mg/mL. **c** Plot of residual OCT content as a function of the initial OCT concentration

Next, the potential involvement of CYP3A4, the cytochrome P450 isoform most commonly involved in drug metabolism in humans, in OCT metabolism was determined. No changes in the activity of recombinant human CYP3A4 were observed when the enzyme was pre-incubated with OCT (400 mM). Thus, the OCT concentration used in this study did not inhibit CYP3A4 activation. An inverse linear correlation was observed between incubation time and residual OCT concentration, where the most effective incubation time was 10 min (Fig. 4a–c). An inverse linear relationship was also observed between recombinant CYP3A4 protein concentration and residual OCT concentration, where the most effective protein concentration was 2.5 mg/mL. Under these conditions, the residual OCT content decreased in line with a decrease in the initial concentration of OCT (*p* < 0.05), and many different metabolites were observed via LC–MS/MS. These results demonstrated that CYP3A4 metabolized OCT.

### P-gp, MRP2, and CYP3A4 expression is increased in the intestinal mucosa of rats with PH

**Fig. 5:**
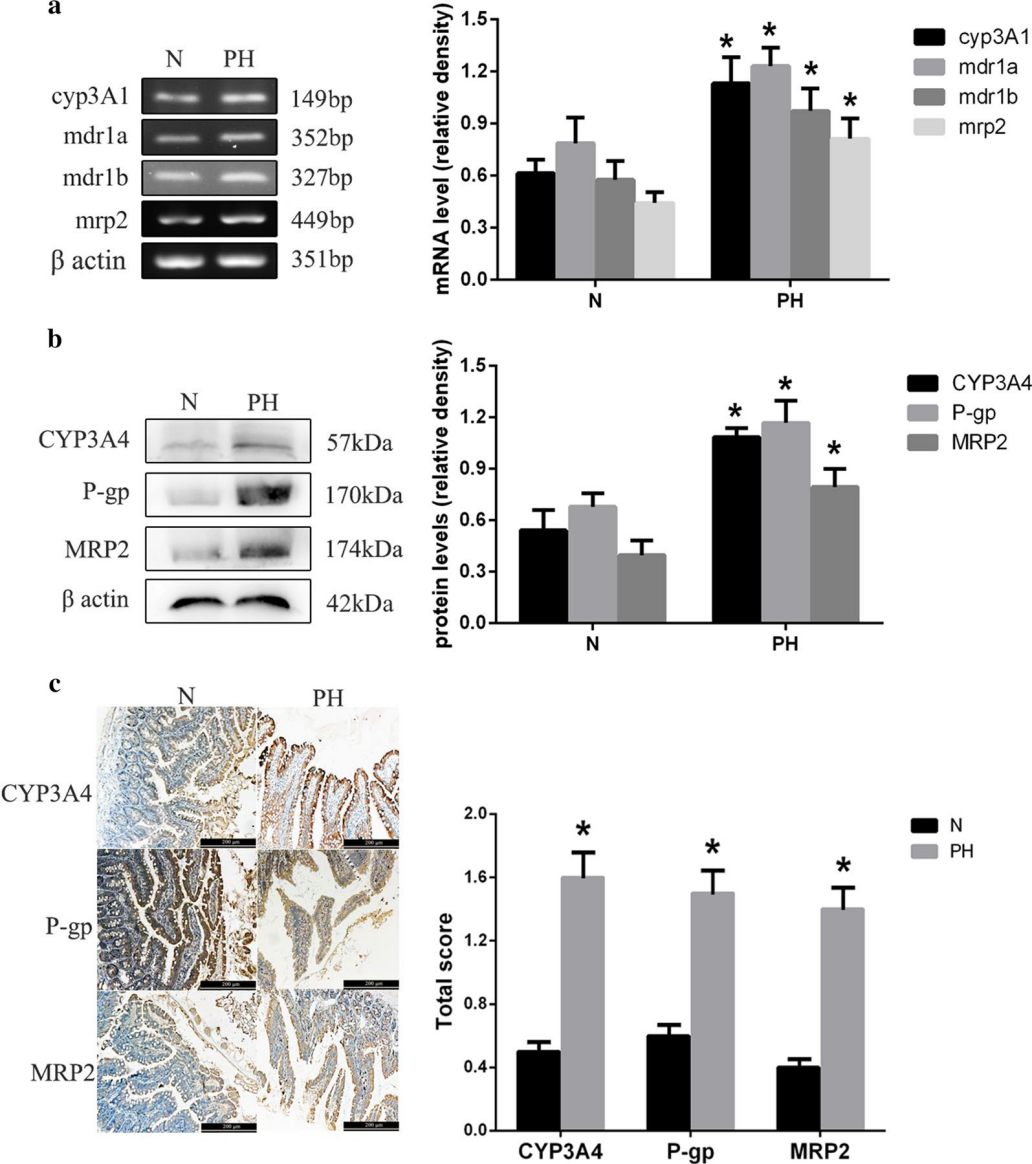
P-gp, MRP2, and CYP3A4 expression in the intestinal mucosa of normal and PH rats. **a** RT-PCR results showing cyp3A1, MDR1 (mdr1a, mdr1b), and mrp2 mRNA expression in the intestines of normal (Group N) and PH (Group PH) rats. **b** Western blot showing CYP3A4, P-gp, and MRP2 protein expression in rat intestine. **c** Immunohistochemical detection of CYP3A4, P-gp, and MRP2 in rat intestine (100x magnification). Results are expressed as mean ± S.D. (*n* = 3). *p < 0.05, Group PH versus Group N

RT-PCR and western blot analysis revealed that P-gp, MRP2, and CYP3A4 mRNA and protein expression was significantly higher in PH rats compared to the normal ones (*p* < 0.05) (Fig. 5a, b). On the other hand, the immunohistochemical analysis revealed significantly higher P-gp/MRP2/CYP3A4 scores in PH samples compared with the scores in the controls (*p* < 0.05) (Fig. 5c). These results demonstrated that P-gp, MRP2, and CYP3A4 were upregulated in the intestinal mucosa of rats with BDL-induced PH.

## Discussion

In this study, the mechanism mediating OCT absorption was studied in a rat model of biliary cirrhosis with PH induced via BDL. This method was used to reduce cholestasis in the intestine, easily inducing PH conditions, and diminishing rat fatality. A previous study showed that the establishment of a portosystemic shunt decreases hepatic first-pass effects under PH conditions [17].

As the intestinal first-pass effect also greatly influences the oral absorption and bioavailability of drugs, our next experiments addressed OCT absorption mechanisms in the jejunum, the main site of OCT absorption in the intestine [47], in both control and PH rats.

P-gp is an important member of ATP binding cassette transporter family (ABC). ABC is an important factor affecting the drug action during the body process i*n vivo*, and P-gp is the most important transporter. MRP is a carrier protein with ABC and plays an important role in the dynamic changes of drugs in vivo. Like P-gp, MRP2 can restrict the absorption of substrates through the intestinal tract, forming an intestinal absorption barrier and is the main target of excretion of drugs through intestine [18–21]. Previous studies showed that P-gp and MRP-2 inhibit the passage of OCT through the blood–brain barrier and proximal renal tubules [21, 42]. Considering that the two efflux transporters can significantly inhibit intestinal absorption of many drugs in the intestine, our hypothesis was that P-gp and MRP-2 might also play an important role in the first-pass intestinal effect of OCT. CYP450 enzyme system is the most important phase I metabolic enzyme in vivo. It participates in the metabolic transformation and elimination of endogenous and exogenous substances, especially CYP3A4. As a major enzyme system, CYP3A4 is involved in the first-pass effect process of oral drugs, which is also an important factor affecting drug-drug interaction [43]. P-gp, MRP2, and CYP3A4 often have overlapping substrates; thus, they have synergistic effects on the same substrate. Therefore, our further speculation was that CYP3A4 might also affect the metabolism and absorption of OCT in the intestinal tract, playing an inhibitory role together with the efflux transporters.

Our in situ jejunal perfusion and everted intestinal sac studies demonstrated that the absorbed OCT concentrations were higher when P-gp and MRP2 inhibitors were co-administered than when each drug was administered alone. In addition, P-gp inhibition had a stronger effect than MRP2 inhibition, suggesting that P-gp was the main driver of OCT efflux in the rat intestine. Intestinal OCT uptake was impaired in PH rats, and this phenomenon could be explained by the increased expression of P-gp, MRP2, and CYP3A, as detected by RT-PCR, western blotting, and immunohistochemistry. A previous study showed that OCT inhibits MRP2 [54], suggesting that MRP2 has minor or no influence on OCT absorption, which is in contradiction with our results. However, our results showed that the inhibitory effect of OCT on MRP2 was weaker compared with the enhanced first-pass intestinal effects of P-gp, MRP2, and CYP3A4 on PH condition, thus inducing mal-absorption of OCT. In addition, our results highlighted that P-gp was the main driver of OCT efflux in the rat intestine, compared to MRP2, thus resulting in a greater effect on OCT absorption, thus being in accordance with the previous study [54].

The involvement of both P-gp and MRP2 in OCT absorption was further demonstrated in Caco-2 cell transport assay. Human colon cancer cell line Caco-2 is a commonly used model for studying drug transporters. Caco-2 cell model has been widely used in the study of intestinal absorption of drug molecules in vitro due to its similar morphological and biochemical properties to intestinal epithelial cells. Previous experiments confirmed that MDR1 and MRP2 are highly expressed in Caco-2 cells [55]. These cells not only reproduce the intestinal drug absorption, but also reflect the permeability of drugs through intestinal transmembrane transport [56]. Therefore, Caco-2 cell line is the ideal model for this experiment.

Our rat intestinal microsome study also suggested an increased CYP3A activity in PH rats than in normal rats. OCT was also found to be metabolized by recombinant human cytochrome P450 3A4 (CYP3A4), in a process blocked by ketoconazole. Ketoconazole inhibits a wide range of CYP enzymes, but at a concentration of 10 µM, like the one used in this work, is selective for CYP3A4 and CYP3A5 [57]. Therefore, our hypothesis was that OCT might be a substrate of CYP3A4.

OCT concentrations absorbed in the everted intestinal sac model were significantly higher than those measured in the in situ intestinal perfusion model. This difference could be due to the absence of a physiological environment, such as lack of peristalsis and intestinal microflora. CYP expression may also change *ex-vivo*, leading to individual differences in pharmacokinetics [58]. Previous studies revealed that changes in efflux transporters and metabolic enzymes occur in both the intestine and liver during liver disease. P-gp expression and CYP isoenzymatic activity in the small intestine are also enhanced in liver fibrosis, contributing to the decreased bioavailability and increased elimination of ofloxacin after oral administration [6]. A previous study also demonstrated that the expression of hepatic efflux transporters is upregulated in liver failure induced by toxic levels of acetaminophen or by primary biliary cirrhosis (PBC). Hepatic efflux decreases the retention of the by-products of cellular injury and bile constituents within hepatocytes. In addition, the increased expression of efflux proteins is a critical response to hepatic damage to protect the liver from additional injury [7]. A study in patients with PBC at an advanced-stage revealed that the export pump MRP2 is preserved and the MDR P-gp (MDR1, MDR3) is increased in canalicular liver plasma membranes, leading to the downregulation of the basolateral uptake systems. The maintenance or upregulation of canalicular and basolateral efflux pumps may represent adaptive mechanisms to limit the accumulation of toxic biliary products [8]. Cholestatic liver disease and increased concentration of serum bile acids are known as triggering various adaptive responses, including the induction of hepatic, intestinal, and renal bile acid transport proteins [9]. Results of a previous study indicated that the increased expression or activity of export pumps and CYP enzymes may occur in the intestine or even in the liver in cases of cirrhosis complicated by PH, which was actually the same discovered in this work. Previous studies primarily focused on investigating P-gp and CYP3A4 inhibition to facilitate drug transport [10, 11, 46]. Our study also revealed that MRP2 contributed to the intestinal first-pass effect on OCT bioavailability in addition to P-gp and CYP3A4. Thus, the inhibition of P-gp, MRP2, and CYP3A4 might be a promising strategy to effectively decrease PH via oral administration of OCT.

Decreased hepatic levels of P-gp, MRP2, and CYP enzymes during the development of injuries such as Child’s class C cirrhosis or PBC III [12–14] have been reported. In general, drug dosages should be decreased in patients with severe cirrhosis [15]. Changes in transporter expression in the liver are also responsible for cholestasis and are the result of various factors. The downregulation of hepatobiliary transport systems is observed in inflammation-induced cholestasis in humans, while transporter expression does not change in anicteric cholestasis during PBC stages I or II [16]. However, no study investigated the potential alteration in P-gp, MRP2, or CYP3A4 expression or activity in the intestine. The BDL cirrhosis model used in our study induced PH via extrahepatic obstruction without severe liver damage, as previously reported [59]. Thus, while previous studies reported alterations in P-gp, MRP2, and CYP3A4 in severe liver injury, hepatic expression and activity of exporters and CYP enzymes may not be significantly altered in the BDL model.

The current study did not address the mechanisms by which P-gp, MRP2, or CYP3A4 expression and/or activity are dysregulated in the intestine of PH rats. The pregnane X Receptor (PXR)-Retinoid X Receptor alpha stimulates CYP3A4 transcription through the activation of the CYP3A4 promoter [60, 61]. P-gp is also regulated by nuclear receptors such as PXR [62], ERK-FOXO 3a [63], while MRP2 is regulated by protein kinase C, radixin, nuclear factor kB, human cAMP response element-binding protein, and the CAATT box enhancer binding protein [64–66]. Further studies are planned to investigate the molecular mechanism underlying the alteration in drug efflux transporters and detoxifying enzymes associated with PH. In addition, we are aware of the difficulty to evaluate a proper oral administration of OCT by P-gp, MRP2, and CYP3A4 inhibition. Some aspects need to be explored in further details, such as the importance of these transporters and enzymes for hepatic/renal excretion of OCT, drug-drug interactions, and appropriate drug-therapy titration.

## Conclusions

In conclusion, our work demonstrated that the decreased oral bioavailability of OCT was potentially related to the increased expression of P-gp, MRP2, and the metabolic enzyme CYP3A4, which increased the intestinal first-pass effect on OCT, thus limiting its anti-portal hypertensive efficacy. Targeted inhibition of P-gp, MRP2, and CYP3A4 might increase OCT oral absorption to effectively treat PH.

## Supplementary information

**Additional file 1: Table S1**. The details of RT-PCR primers.

## Data Availability

The datasets used and/or analyzed during the current study are available from the corresponding author on reasonable request.

## Abbreviations

PH: Portal hypertension
OCT: Octreotide
BDL: Bile duct ligation
P-gp: P-glycoprotein
MRP2: Multidrug resistance-associated protein 2
CYP3A4: Cytochrome P450 3A4
KRB: Krebs–Ringer bicarbonate buffer
AUC: Area under curve
TEER: Transepithelial electrical resistance
PDR: Permeability direction rate
PPB: Potassium phosphate buffer
RT-PCR: Reverse transcription-polymerase chain reaction
RIPA: Radio-immunoprecipitation assay
MRM: Multiple reaction monitoring
PBC: Primary biliary cirrhosis
PXR: Pregnane X Receptor
