# Supplementary material for: Overexpression of P-glycoprotein, MRP2, and CYP3A4 impairs intestinal absorption of octreotide in rats with portal hypertension
Source: BMC Gastroenterol. 2021 Jan 6;21:2. doi: 10.1186/s12876-020-01532-4 (PMC7789354; doi:10.1186/s12876-020-01532-4)
Supplement: Supplementary file 1 — Additional file 1: Table S1. The details of RT-PCR primers. [file 12876_2020_1532_MOESM1_ESM.docx]

Table S1 RT-PCR primers

F, forward; R, reverse
